# Improved image contrast in nonlinear light-sheet fluorescence microscopy using i^2^PIE Pulse compression

**DOI:** 10.1038/s41598-024-63429-6

**Published:** 2024-06-04

**Authors:** Imraan Badrodien, Pieter H. Neethling, Gurthwin W. Bosman

**Affiliations:** 1 Stellenbosch Photonics Institute, Physics Department, Stellenbosch University, Stellenbosch, Western Cape South Africa; 2 National Institute for Theoretical and Computational Sciences (NITheCS), Stellenbosch, South Africa

**Keywords:** Light-sheet fluorescence microscopy, Nonlinear microscopy, Biophotonics, Applied optics, Light-sheet microscopy, Multiphoton microscopy

## Abstract

Nonlinear microscopy has become an invaluable tool for biological imaging, offering high-resolution visualization of biological specimens. In this manuscript, we present the application of a spectral phase measurement technique, i^2^PIE, to compress broad-bandwidth supercontinuum pulses for two-photon excitation fluorescence light-sheet fluorescence microscopy. The results demonstrated a significant improvement in the two-photon excitation response achieved. We also showed that the implementation of i^2^PIE allowed for enhanced image contrasts when compared to conventional compression techniques, with i^2^PIE producing an image contrast improvement over conventional methods by over 50%.

## Introduction

Light-sheet fluorescence microscopy (LSFM) has allowed for faster three-dimensional imaging of biological samples with lower photo-bleaching and photo-toxicity than confocal microscopy^1^. This is achieved by decoupling the illumination and detection axis and restricting the excitation of fluorophores to the imaging plane^2^.

The use of nonlinear optical processes has become ubiquitous in microscopy applications. One such process is two-photon excitation fluorescence (2PEF) which allows for the use of near-infrared ultrashort pulse illumination sources that offer deeper penetration depths and reduced out-of-focus fluorescence due to the restriction of excitation to the focal plane^3^. Ultrashort laser sources provide the necessary high peak intensities that induce nonlinear responses, however, care must be exercised as photodamage may result in the case of excessive average powers^4^. In addition, 2PEF in digitally scanned light-sheet microscope (DSLM) configurations have provided an ideal technique to image large fields of views with improved image contrast, but again at the cost of higher average powers^5^. Thus for optimal fluorescence detection and subsequent imaging, maintaining the incident radiation below the damage threshold of biological samples is therefore a key concern when implementing nonlinear microscopy techniques^6^.To increase the nonlinear 2PEF signal, while maintaining an average power below the damage threshold, the pulse repetition rate could be reduced and the pulse energy increased^7,8^, or simply by decreased the pulse duration^7,9^. In this paper, we will implement the latter, ensuring that the pulse duration at the sample plane is its so called bandwidth-limited duration. This bandwidth-limited pulse duration implies that any dispersion that may lead to temporal pulse broadening needs to be accounted for before the pulse reaches the sample plane. The optimal pulse compression of the incident pulse enhances nonlinear processes without changing the linear effects such as photodamage^10^.

Recent advances in the development of all-normal-dispersion photonic crystal fibres (ANDi-PCF), have allowed for the generation of highly stable broadband supercontinuum (SC) pulses, with uniform and flat spectral profiles, which can be temporally compressed^11^. By incorporating a 4f pulse shaper with a one-dimensional spatial light modulator (SLM), compression of the SC pulses can be achieved by applying appropriate phase masks to the SLM. In this regard, multiphoton intrapulse interference phase scanning (MIIPS) has become a popular choice for pulse characterisation^12–14^, but it is limited by its ability to only consider second-order dispersion corrections, and due to the time-consuming incremental phase reconstruction process^15^.

An alternative technique to characterize ultra-short pulses, i^2^PIE, was proposed and developed by Spangenberg et al.^16^. This technique is an extension of the time-domain ptychographic iterative engine (PIE) to generalized spectral phase-only transfer function, and its ability to reconstruct the amplitude and phase of a pulse has been demonstrated^16^. The i^2^PIE technique requires only a single spectrogram as input. This is done by measuring the spectra while sequentially applying phase-only transfer functions to an unknown signal and recording the resultant second-harmonic spectrum. The iterative engine is then applied to the spectrogram to reconstruct the spectral phase. This technique can determine higher-order contributions, and due to the single input, converges fast and reliably. The capabilities of this technique have already shown promise for both spectroscopy and microscopy applications^17,18^. The applications of the i^2^PIE technique in nonlinear confocal microscopy have already been demonstrated and showed improved contrast and signal-to-noise ratios when compared to other pulse compression techniques^17^.

In this paper, we demonstrate the use of a broadband SC pulse, generated in an ANDI-PCF, and compressed using the i^2^PIE phase measurement technique for imaging in nonlinear 2PEF light-sheet microscopy. The performance of the i^2^PIE aided pulse compression technique applied to 2PEF light-sheet microscopy is compared to an uncompressed pulse (the fundamental pump pulse) and SC pulses compressed using a combination of MIIPS and compression employing chirped mirrors. Finally, we illustrate the application of this new 2PEF light-sheet imaging method when imaging the cellular nuclei of cardiomyoblast cells arranged in a spheroid.

## Results

### Phase measurements and pulse compression

**Figure 1:**
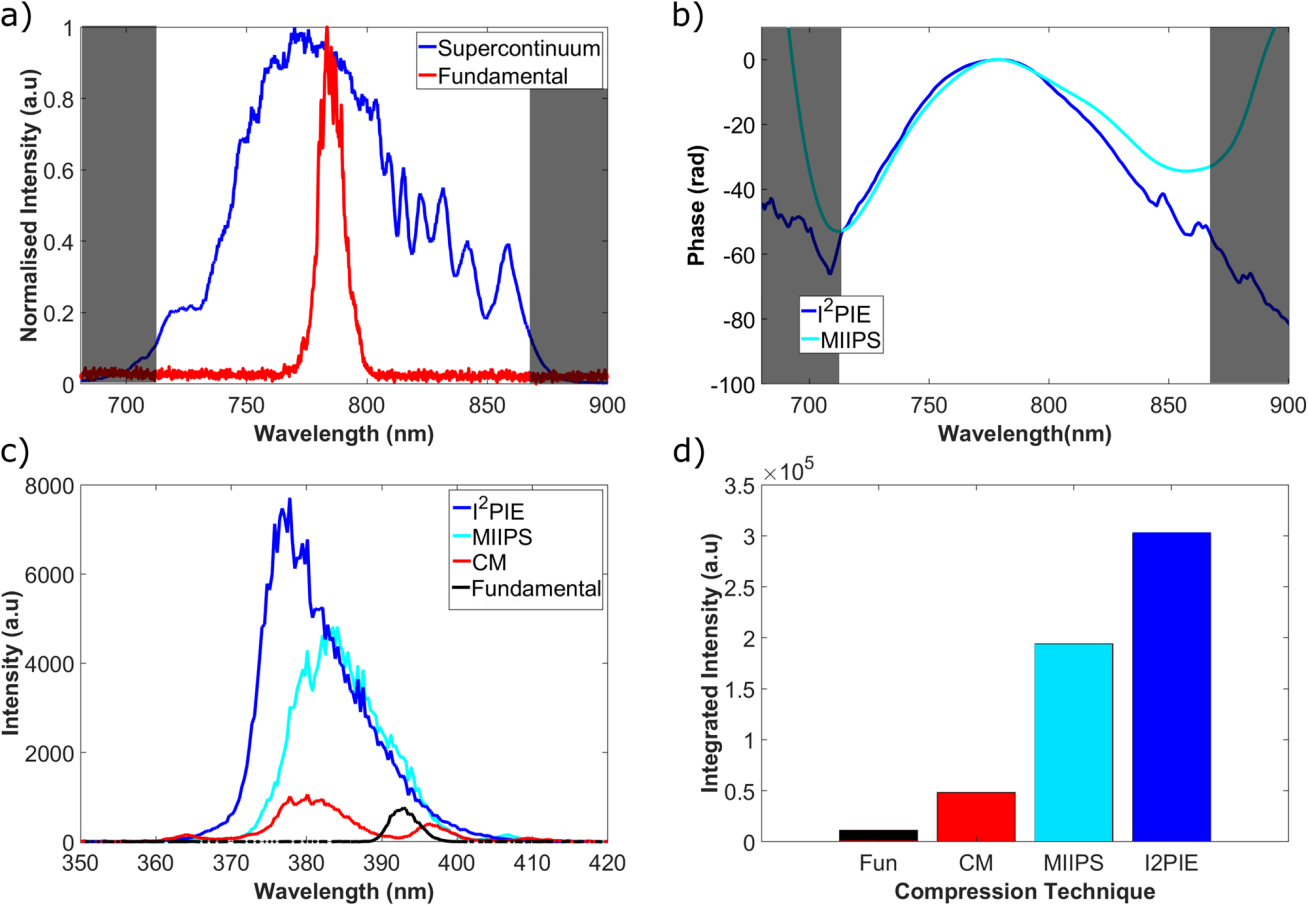
(**a**) The measured spectra of the fundamental pump pulse and the generated broad bandwidth SC pulse from the ANDi-PCF. (**b**) The measured spectral phases obtained using the i^2^PIE and MIIPS phase retrieval techniques for the system. (**c**) The recorded second-harmonic generation spectra from a BBO crystal placed at the sample plane for each compression technique. (**d**) The integrated second-harmonic generation response from the spectra in (**c**).

The broad bandwidth SC pulse was generated through optically pumping an ANDI-PCF with a (fundamental) transform-limited (TL) ultrashort laser pulse with a center wavelength of 785 nm and a full-width half maximum (FWHM) bandwidth of 12 nm. The fibre was pumped with an average power of 700 mW, with an average output power from the fibre of 420 mW. The measured SC and fundamental spectra are shown in Fig. 1a. The bandwidth of the SC pulse was determined to be 160 nm, spanning the range from 710 to 870 nm, as depicted in Fig. 1a. Assuming that this SC pulse is transform-limited at the sample and has a Gaussian intensity temporal profile, one calculates that the duration of the TL pulse when fully compressed is on the order of 6 fs. Unfortunately, achieving this duration is challenging due to the fibre’s nonlinear dispersion and the presence of additional optical elements before the sample.

To determine the spectral phase of the supercontinuum, a beta-barium borate crystal (BBO) crystal is placed at the sample plane with the emitted second harmonic generation (SHG) spectrum recorded using a spectrometer. By measuring the spectral phase at the sample plane of the microscope, the dispersion of all the optical elements along the beam path is accounted for. Since BBO crystals are hygroscopic, the sample chamber was removed and a cuvette of water was introduced before the illumination lens. This cuvette would then be removed once the phase measurement was completed and the sample chamber would be inserted at the sample plane. This allowed for an acceptable compromise that approximated the dispersion of the sample which was mounted in agarose and suspended in water. An alternative method to this would be to compute the dispersion that resulted from the introduction of the sample, and to add an appropriate phase onto the measured spectral phase acquired with one of the phase retrieval techniques, however this was not implemented in this study.

The two phase measurement techniques, i^2^PIE and MIIPS were implemented, and their measured spectral phases are shown in Fig. 1b. The measured spectral phases showed the same general shape within the region of interest, however the phase measurement obtained with i^2^PIE showed more structure than the phase acquired using MIIPS. The MIIPS phase measurement technique, while able to measure quadratic phases, was unable to accurately measure higher-order contributions resulting in an overall smoother phase measurement. In contrast, the finer details observed in the phase measurement using i^2^PIE indicated a higher sensitivity to higher-order contributions.

By applying the negative of these measured phases, the supercontinuum pulses were compressed. The changes in the generated second harmonic (SH) responses from the BBO crystal for each phase measurement technique were recorded using a spectrometer and are presented in Fig. 1c. The SHG response from the BBO crystal for the fundamental and supercontinuum using only chirped mirrors for compression is included as a reference. The SH signal in the spectrometer was optimized for both the broadest and strongest signal. From the recorded spectra, the use of the supercontinuum source produced a broader SH spectrum than the fundamental. Additionally, applying pulse compression using either compression technique, produced a broader spectrum with higher peak intensities. Assuming the phase applied to the SLM only shapes the excitation pulse temporally, the measured SH spectra were integrated as a measure of the total SH response in the BBO crystal as shown in Fig. 1d. The SH response obtained with the compressed SC using the i^2^PIE and MIIPS phase measurements produced a 6 and 4-fold improvement respectively when compared to the SC compressed with only the chirped mirrors.

### Two-photon excitation fluorescence

**Figure 2:**
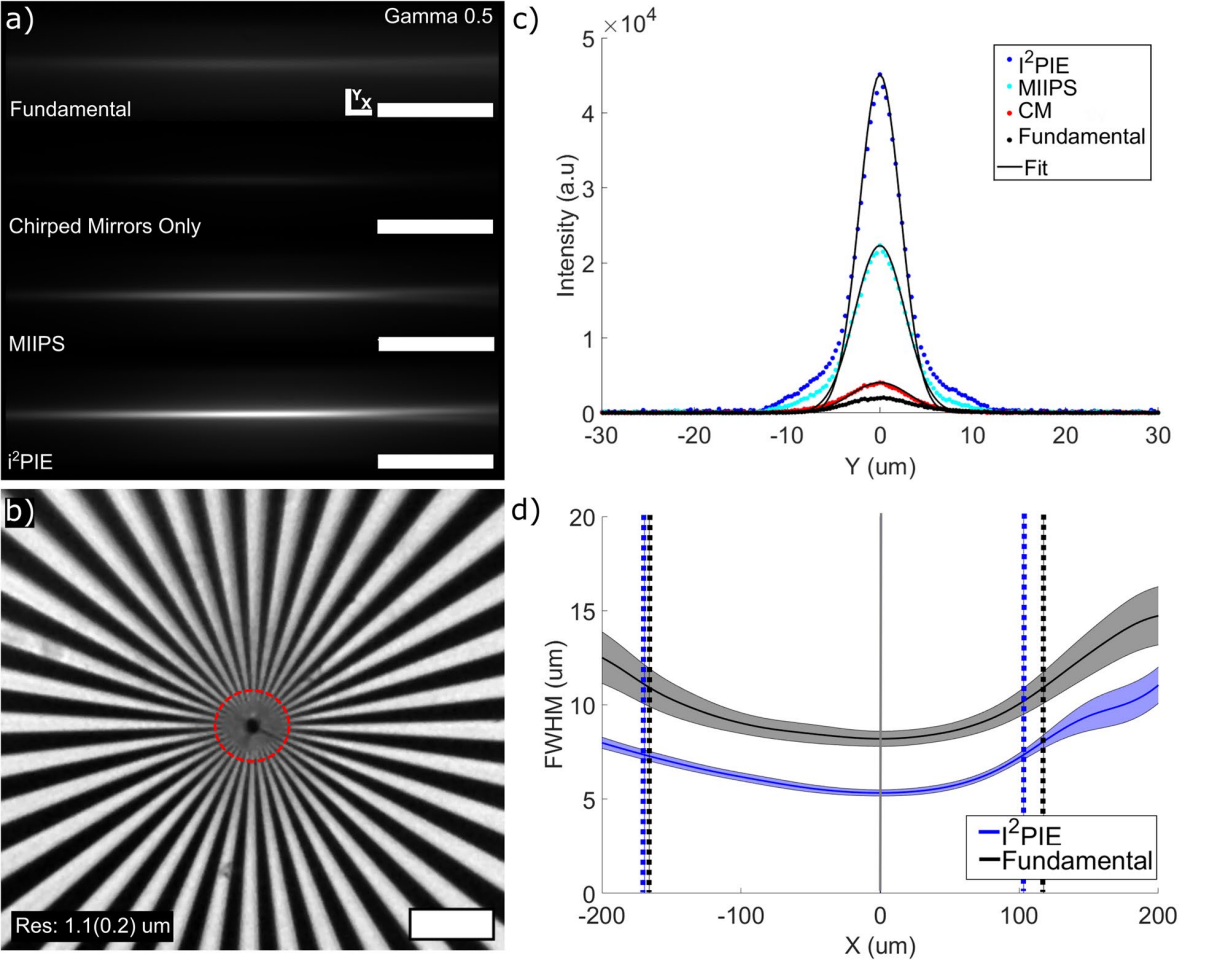
(**a**) The two-photon excitation fluorescence images of a Gaussian beam in Coumarin-102 fluorescent dye for the different pulse compression techniques (Scale bars represent 100 $\mu$m). (**b**) Imaged Siemans star used to determine the transverse resolution of the imaging system. (**c**) Examples of vertical line-outs near the focal plane of the beam with Gaussian fits used to determine the FWHM of the sheet at each position along the propagation axis. (**d**) Evolution of the FWHM waist around the focal plane of the sheet for the fundamental and supercontinuum compressed with i^2^PIE, from which the light-sheet thickness and field of view were obtained.

The following set of measurements was performed to characterize the light-sheet parameters of the microscope system. The dimension of the two-photon excitation fluorescent beam profiles was determined by imaging the emission profile of the beam in the Coumarin-102 fluorescent dye dissolved in ethanol. For this measurement, the galvanometer mirror was set to a stationary position and images were acquired for each compression technique. Images were acquired with an average excitation power of 40 mW at 1 s exposure and are presented in Fig. 2a. A gamma filter of 0.5 was applied to the image acquired using the uncompressed, narrow bandwidth fundamental pulse to make the features visible. In light-sheet imaging, the axial resolution is determined by both the light-sheet thickness and the imaging system, while the transverse resolution is determined almost entirely by the imaging system^19^. The transverse resolution (xy plane) of the imaging system was determined by imaging a Siemens star target shown in Fig. 2b. From the imaged star, the average spacing between dark and light spokes can be determined by obtaining the intensity profile along the edge of a circle. The radius of the ring is decreased until the features are just resolvable and this was taken as an indication of the transverse resolution of the system, where the resolution was determined as 1.1 ± 0.2 $\mu$m, where the uncertainty is the standard deviation.

Since the axial resolution is controlled by the sheet width, vertical line-outs, showing the intensity profile along the y-axis, were taken at each pixel position along the horizontal axis of each image in Fig. 2a and a Gaussian function was fitted to the data from which the full width half max (FWHM) beam thickness at each position along the horizontal axis was acquired. Examples of the vertical line-outs near the focal plane are shown in Fig. 2c. From these intensity profiles, the peak intensity profiles using the supercontinuum with only the chirped mirrors produced a 2$\times$ improvement in the peak intensity over the fundamental beam. The peak intensity near the focal plane for the compressed pulses using the i^2^PIE and MIIPS phase measurement techniques produced an 11$\times$ and 5$\times$ improvement respectively over the supercontinuum with only the chirped mirrors.

Figure 2d presents the evolution of the FWHM widths of the beam along the propagation axis for the fundamental and the supercontinuum compressed using i^2^PIE. A spline fit was applied to represent the data as there is an asymmetry around the beam waist. This is due to a combination of chromatic aberrations and the nonlinear refractive index of the medium, which are amplified due to the large range of wavelengths present in the supercontinuum and the increased probability of nonlinear processes due to the increase in peak intensities as a result of pulse compression. The effect of chromatic aberration was minimised by using a low NA achromatic lens for illumination^20^. From the evolution of the FWHM thickness of the beam profiles, the light-sheet FOV was determined as the distance between the two points around the beam waist where the sheet thickness increases by a factor of $\sqrt{2}$ as indicated in Fig. 2d by the vertical lines.

The light-sheet parameters are presented in Table 1. As expected, due to the increased intensity (or peak power), the compressed pulses resulted in a thinner sheet thickness along with a corresponding decrease in the FOV of the sheet.

**Table 1:** Sheet parameters for the DSLM system using Gaussian beam profile, where the uncertainties were determined by the fits.

Excitation	Sheet thickness ($\upmu$m)	FOV ($\upmu$m)
Fundamental	7.7 ± 0.5	283 ± 45
CM Only	6.9 ± 0.2	364 ± 51
MIIPS	6.4 ± 0.1	317 ± 30
i^2^PIE	5.2 ± 0.1	272 ± 19

### Imaging spheroids of cardiomyoblast cells

**Figure 3:**
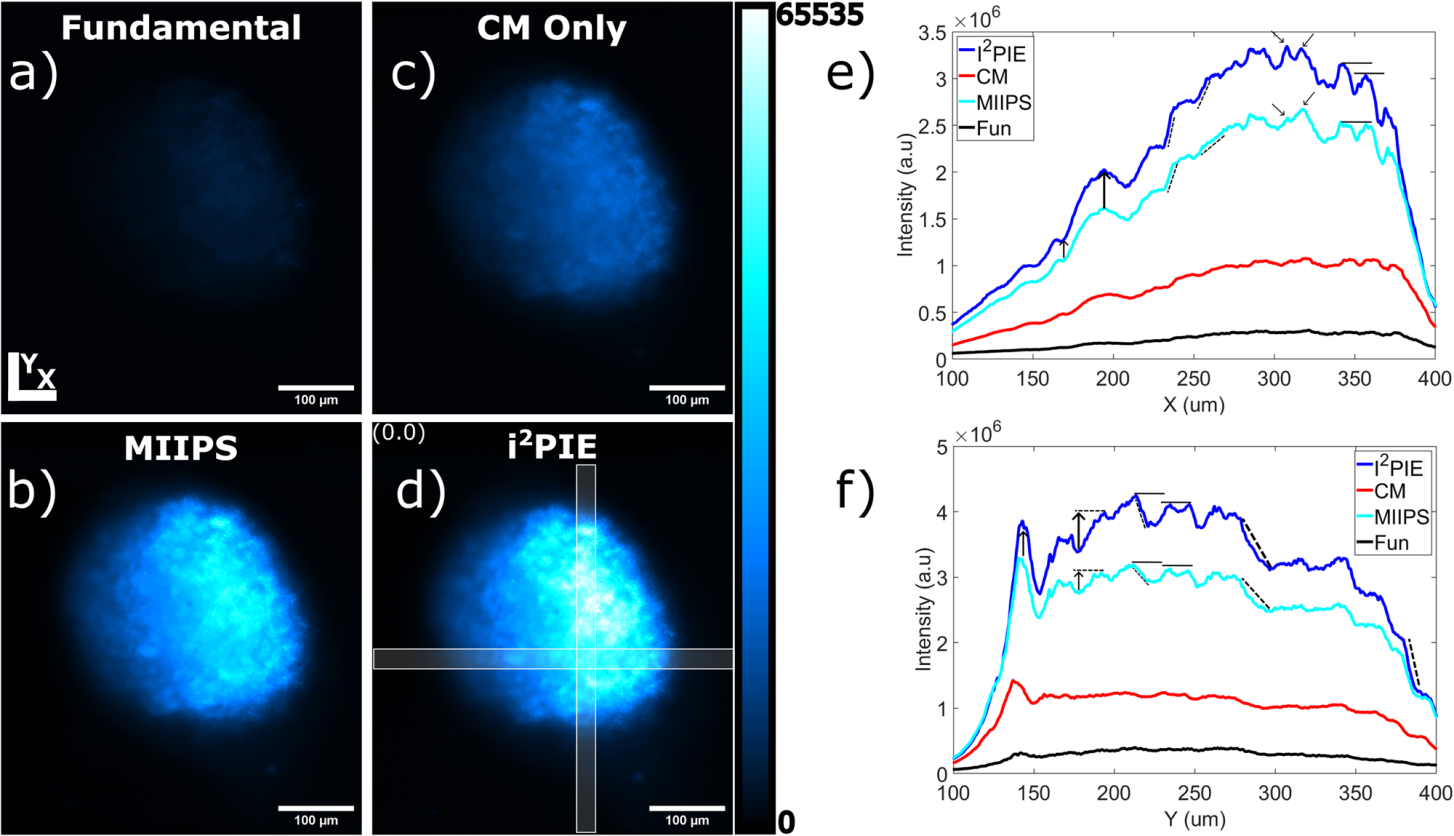
2PEF imaged sections of the sample plane of a spheroid of cardiomyoblast cells. (**a**–**d**) The imaged planes using different excitation techniques. (**e**,**f**) Average horizontal and vertical line-outs for each excitation technique showing contrast improvements for each technique. Selected regions shown in (**d**).

Single planes of a spheroid of cardiomyoblast cells were imaged using each compression technique. The nuclei of each cell stained with Hoechst 33342 served as an ideal 2PEF source. The images were acquired at an average illumination power of 40 mW with an exposure time of 2 s. The unprocessed images (raw images, 16 bit) acquired are shown in Fig. 3a–d. From the images, there is a visual improvement in the two-photon signal strength when pulse compression is performed using the phase measurement techniques.

The image intensity values were taken as an indication of the two-photon response in Fig. 3a–d. The intensity values in a 10 $\mu$m $\times$ 10 $\mu$m region around the maximum of i^2^PIE was integrated and compared with the corresponding regions of the other images as indicated in Fig. 3d. The improvements of i^2^PIE demonstrate that by decreasing the pulse duration, stronger two-photon responses could be achieved at a fixed average power. The use of the broadband SC pulse compressed with the i^2^PIE technique produced nearly a 12$\times$ improved two-photon response in the spheroid over the fundamental pulse. In addition, the i^2^PIE technique also produced a 4$\times$ and 1.5$\times$ improved two-photon response over the SC compressed using only chirped mirrors and the SC compressed with the MIIPS technique. Since the two-photon response scales quadratically with the incident intensity, the fundamental would require approximately 3.5$\times$ more average power (> 140 mW) at the sample to achieve an equivalent response to that of i^2^PIE. Conversely, when imaging these samples, the application of broadband SC together with i^2^PIE pulse compression could thus achieve similar results to that of a typical femtosecond laser source but at just 30$\%$ of the incident average power.

Optimising the two-photon fluorescence signal requires maintaining the pulse energy at the sample below the saturation energy^21^. As discussed by Charan et al.^10^ the saturation energy can be estimated from the peak excitation wavelength ($\lambda = 785$ nm), pulse duration ($\tau = 76$ and 6 fs for the fundamental and SC compressed with i^2^PIE respectively), the NA of the light-sheet (NA = 0.15) and the two-photon absorption cross section of Hoescht-33342 ($\sigma _{2} = 5$ GM^22^). The saturation energy for our system ranges between 12 nJ (SC compressed pulse) and 44 nJ (fundamental pulse). We performed the measurements at pulse energies of approximately 500 pJ, which ensured that we were sufficiently below the saturation energy. This allowed us to image the samples without potential bias from bleaching and photodamage effects, while demonstrating how i^2^PIE can allow for optimised two-photon response at lower average powers than what would typically be required using standard femtosecond laser sources.

As expected, using the compressed pulses produced the strongest two-photon response due to the increase in peak intensity associated with compressed pulses. Integrated line-outs along the x and y regions of interest indicated in Fig. 3d were acquired for each of the images and are shown in Fig. 3e,f. The increase in the two-photon response is clear as seen by the increase in peak intensities. While similar structures can be seen in the profiles of each excitation method, the contrasts of each begin to become evident. The increase in contrast of i^2^PIE can be seen by the increased gradient of transitions between different features in the profiles, as well as the increased difference between the peaks and the local minimum around different features.

To quantify the contrast improvements seen, the contrast of each unprocessed image was calculated and is presented in Table 2, where the contrast improvements are shown as ratios with respect to the contrast in the images obtained using the fundamental excitation beam. The contrasts were calculated using the Weber contrast definition as defined in the Methods and materials. All contrasts were calculated on the unprocessed images to avoid introducing any bias that may arise during deconvolution and image processing. The uncertainty associated with each value was determined using error propagation.

**Table 2:** Image contrasts of the unprocessed images of the spheroids using different excitation modalities.

Excitation	Image contrast	Improvement ratio
Fundamental	5.7 ± 0.3	1
CM Only	24.6 ± 0.1	4.3
MIIPS	61.5 ± 0.4	10.9
i^2^PIE	93.4 ± 0.5	16.5

From the contrast measurements in Table 2, the excitation using i^2^PIE produced the highest image contrast. When comparing the image contrast of i^2^PIE with MIIPS we find that the percentage contrast improvement is a sizeable 52% . The use of i^2^PIE for pulse compression is therefore able to produce higher two-photon excitation fluorescence responses with improved image contrast over the fundamental femtosecond laser source or the SC pulse compressed with only chirped mirrors or the MIIPS phase measurement technique.

**Figure 4:**
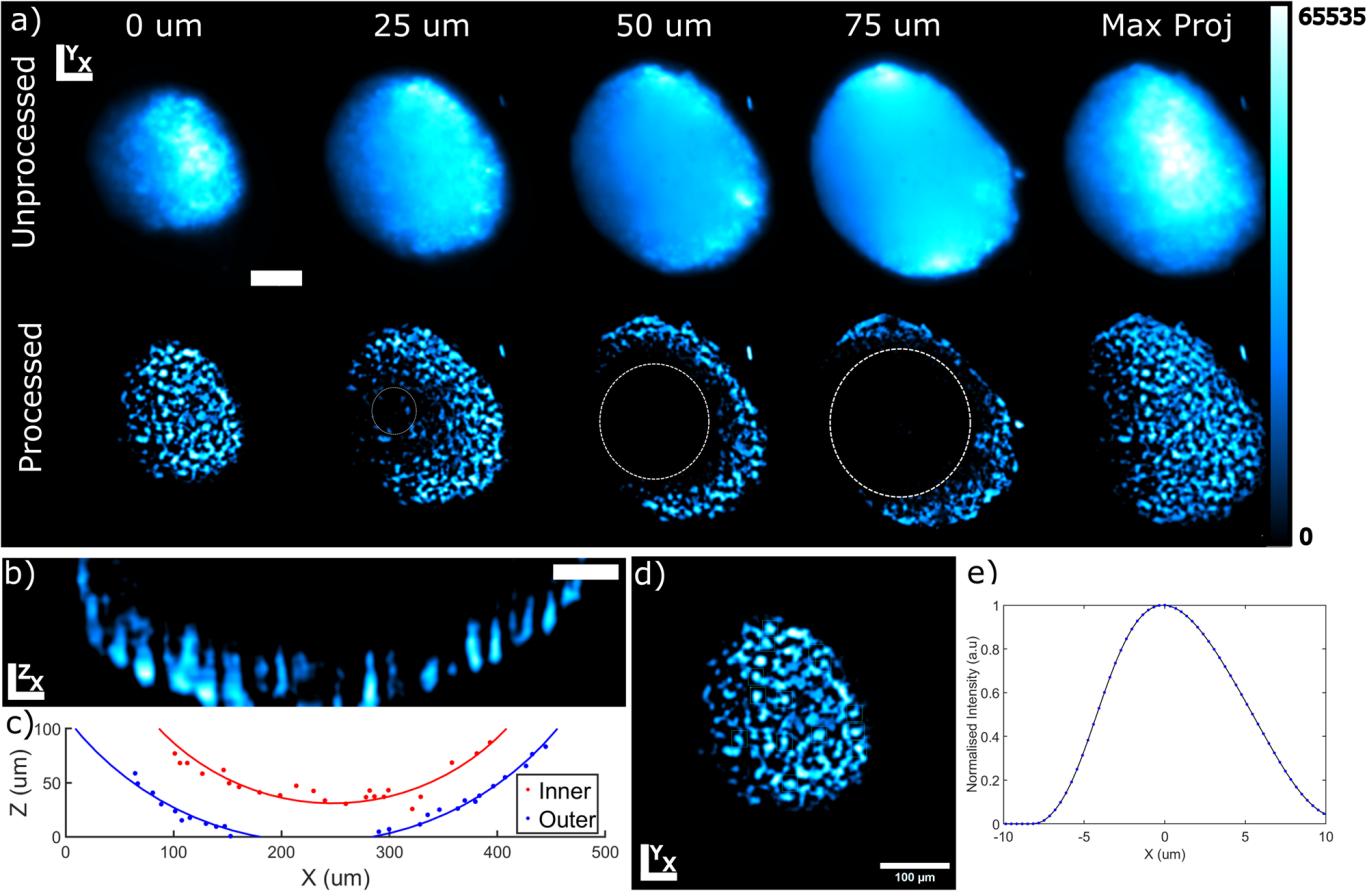
2PEF images of the spheroid of cardiomyoblast cells obtained using the broadband SC pulse compressed using the spectral phase measured with i^2^PIE. (**a**) Processed and Unprocessed images at different depths in the spheroid. Sections were selected at approximately 25 $\mu$m intervals. (scale bar 100 $\mu$m). (**b**) Resliced projection of the spheroid as seen in the xz-plan.(scale bar 50 $\mu$m). (**c**) Circular fits through the inner and outer-surface of the spheroid image. (**d**) Example section of the spheroid where individual nuclei where selected from which there sizes were determined (scale bar 100 $\mu$m). (**e**) Normalised intensity profile of a single nuclei identified from which the FWHM size of each nuclei was determined.

To demonstrate the capabilities of the light-sheet microscope using the broadband supercontinuum with i^2^PIE phase measurements for pulse compression, sectioning through the spheroid was done at 5 $\mu$m steps. The processed and unprocessed sections selected at 25 $\mu$m intervals are shown in Fig. 4a. From these images, individual cell nuclei become visible after processing. The emergence of a dark region at the centre of each section of the spheroid becomes evident at the 25 $\mu$m slice. This region, identified as the necrotic core of the spheroid, continues to increase in size at deeper depths into the spheroid^23,24^.

By re-slicing the full image stack, the spheroid can be visualised from the top as shown in Fig. 4b. From this image a shell-like surface can be seen. By assuming the spheroid to be perfectly spherical, points along the inner- and outer-surfaces of the spheroid were selected and circles were fitted to determine radius of the inner and outer shells of the spheroid as shown in Fig. 4c. The total radius of the spheroid was determined to be 294 ± 25 $\mu$m, with the necrotic region found to have a radius of 224 ± 36 $\mu$m. The necrotic region of the spheroid under these assumptions therefore occupies approximately 44% of the total volume of the spheroid.

Individual nuclei could also be identified at different depths in the spheroid as shown in Fig. 4d. By identifying a total of 55 individual nuclei and selecting regions around the identified nuclei, the averaged intensity profiles of each nuclei were obtained, from which the FWHM of the nuclei were obtained as shown in the example in Fig. 4e. The average FWHM size of the nuclei were found to be approximately 10.95 ± 2.13 $\mu$m, which are expected for mammalian cells^25^.

## Conclusions

We have successfully developed a two-photon light-sheet microscope featuring a broad bandwidth supercontinuum source utilising the novel i^2^PIE spectral phase measurement technique for pulse compression. The use of a supercontinuum pulse allowed for control of the laser pulses to achieve shorter pulse durations than the pulses emerging from the all-normal dispersion fibre. By compressing the pulse durations, higher peak intensities can be achieved in the sample while maintaining the average power at the sample plane. These higher intensities are required to drive nonlinear optical processes, resulting in stronger two-photon excitation fluorescence signals.

When measuring the spectral phases of the supercontinuum, the i^2^PIE algorithm allowed for more accurate measurements over the popular MIIPS technique. The MIIPS technique is restricted in its ability to measure second-order phases, while the i^2^PIE technique was able to account for higher-order phase contributions. The improved accuracy in the phase measurements allowed for shorter pulse durations to be achieved at the sample plane.

Finally, the application of the i^2^PIE technique in two-photon light-sheet fluorescence microscopy has demonstrated improved two-photon excitation fluorescence signals and improved image contrast. When compared to other techniques, i^2^PIE can be used at relatively low average powers and achieve sufficient results.

## Methods and materials

### Experimental setup

**Figure 5:**
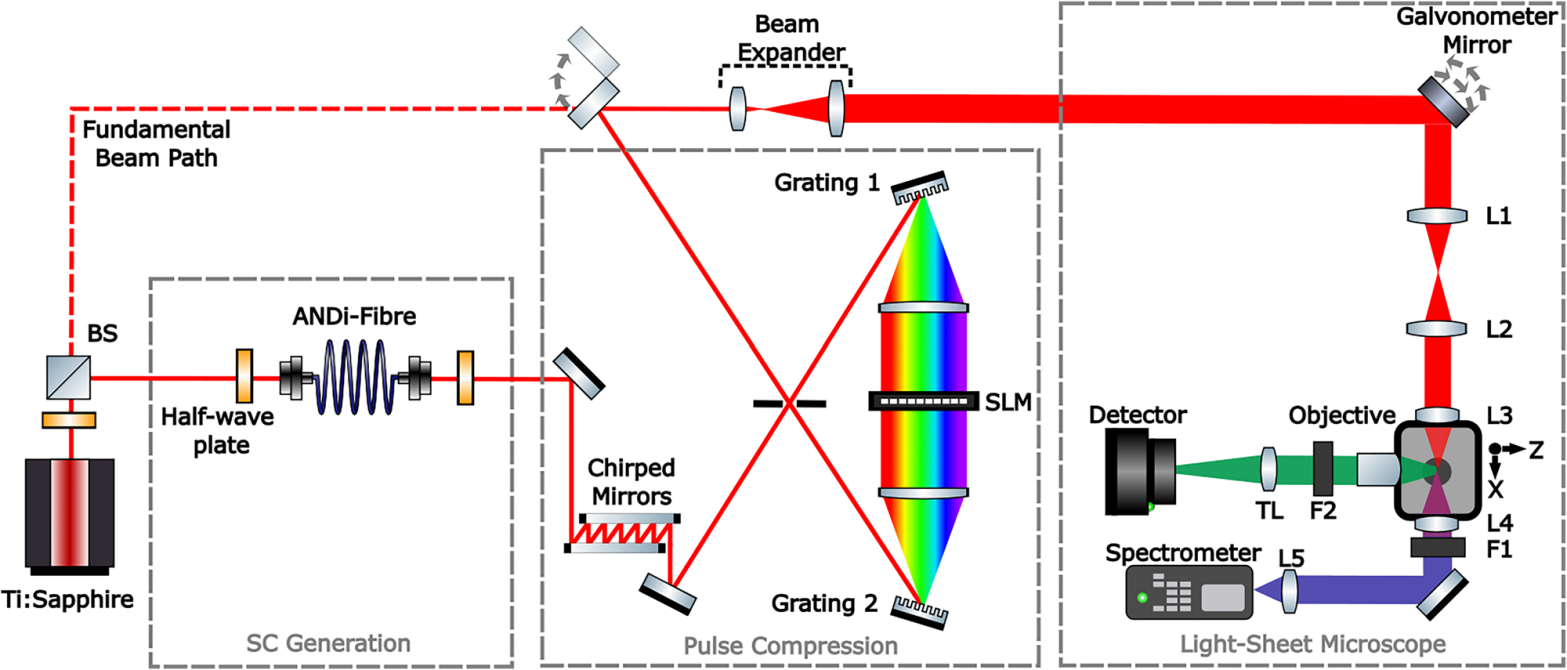
Diagram of the 2PEF light-sheet microscope setup. The setup comprises three parts: supercontinuum generation, phase measurement and pulse compression, and the light-sheet microscope system. The titanium sapphire laser source acts as the pump to generate a broadband supercontinuum source in the all-normal dispersion photonic crystal fibre (ANDi-PCF). Phase measurements are performed at the 4f shaper using a one-dimensional spatial light modulator. The measured phase is used to achieve compressed pulses at the sample plane. A flip mirror and beam expander allows for the selection of either the fundamental or the supercontinuum beam to be used for excitation in the light-sheet microscope system. A beam expander is used to match the sizes of the fundamental and supercontinuum beams. The galvonometer mirror is used to scan the beam along a single axis to produce a light-sheet at the focal plane.

The optical setup implemented in this work is shown in Fig. 5. The system consists of three sections: supercontinuum (SC) generation, phase measurements and pulse compression, and the light-sheet microscope.

The broadband supercontinuum was generated by pumping an all-normal dispersion photonic crystal fibre (NL-1050-PM-NEG, NKTPhotonics) with a tunable femtosecond titanium-sapphire laser (Spectra-Physics, Tsunami) with a central wavelength of 785 nm (12 nm bandwidth) and an 80 MHz repetition rate. Following the generation, the SC was precompressed at the chirped mirrors (Thorlabs DCMP175) to mitigate second-order dispersions. This beam was then directed through a 4f shaper, comprising two gratings, two cylindrical lenses, and a one-dimensional liquid crystal spatial light modulator (SLM).

The excitation beam emerging from the 4f shaper was then sent through the light-sheet microscope system. A flip mirror was used to select the excitation source between the fundamental pump beam or the supercontinuum beam. A beam expander was inserted when using the fundamental beam so that the size of the fundamental matched the size of the supercontinuum to ensure the NA for all beams were the same. The light-sheet microscope system operates on the digital scanned light-sheet microscope (DSLM)^26^. The propagation axis of the excitation beam was defined as the x-axis, with the y-axis along the vertical axis such that the generated light-sheet and imaging planes were in the xy-plane. The translation axis of the specimens were along the z-axis. The light-sheet was formed at the sample plane by and rapidly scanning the excitation beam focused at the sample plane by the achromatic illumination lens L3 (Thorlabs AC127-019-AB-ML). By illuminating only half the back aperture of the lens, we reduced the diffraction effects and ensured a low NA lens. By using an achromatic lens with a low NA as the illumination objective we were able to minimise the effects of chromatic aberration.

The imaging axis, placed along the z-axis, consisted of an objective (Mitutoyo 20X 0.42NA) and a tube lens (Thorlabs TTL200) to produce the desired magnified image on the detector (Hamamatsu Orca Flash 4.0 V2). An emission filter was inserted to separate the excitation and emission wavelengths (Thorlabs FESH0500 and FESH0450).

A second detection axis was also included along the excitation path. This system was used to record the second-harmonic spectra from a beta-Barium borate crystal (BBO) placed at the sample plane when the phase measurements are done. This system consisted of a collection lens L4 (Thorlabs AC127-019-AB-ML) and a shortpass filter to block the red excitation wavelengths and transmit only the second harmonic signal. The collected light is then focused in the spectrometer (AvaSpec-3648, Avantes) to record the second harmonic spectra.

### Phase measurements and pulse compression

Both phase measurement techniques iteratively determine the SC phase at the sample plane to achieve compressed pulses at the sample plane. Both the MIIPS and i^2^PIE techniques use identical setups as shown in Fig. 5, and only a change in software is required to switch between the two techniques.

A 10 $\mu$m thick BBO crystal is placed at the sample plane. The phase measurements for i^2^PIE and MIIPS were done by collecting the second harmonic signal generated in the BBO crystal at the sample plane with the spectrometer. Since the phase measurements are dependent on the system, it is important to account for any dispersions that might be introduced when imaging is done. Biological samples are often suspended in media or water when imaging is done and the introduction of the sample and suspension medium will also cause dispersions and reduce the accuracy of the phase measurements. To account for this, a cuvette containing the appropriate media was placed before the sample plane before the phase measurements were done. This cuvette along with the BBO crystal was removed once the phase measurements were done. The phase measurements and pulse compression was done using custom LabView code.

#### i^2^PIE phase measurement

The i^2^PIE method utilises a spectrogram $S_{n}(\omega )$, with spectrogram number *n*, obtained by multiplying an initial estimate electric field $E_0(t)$ with a known quadratic phase phase-only transfer function,

1 $$\begin{aligned} H_{n}(\omega ) = e^{i \omega ^2 q s_{n}}, \end{aligned}$$

with $\omega$ as the angular frequency and the quadratic phase on the SLM *q* at each step *s*. The spectrogram is produced by Fourier transforming the product of the object function $O_{n}(t)$ with itself, generating frequency-doubled light:

2 $$\begin{aligned} S_n(\omega ) = |\mathscr {F}{(O_n(t))}^2|^2. \end{aligned}$$

After taking the Fourier transform of the recorded spectrum and applying the time domain ptychographic iterative engine algorithm (PIE)^16^, a new object function $O'_{n}(t)$ is obtained. The unique step in the i^2^PIE algorithm is then to update the current electric field estimation to generate a new electric field:

3 $$\begin{aligned} E(\omega ) = O'_{n}(\omega )H^*_{n}(\omega ), \end{aligned}$$

where $O'_n(\omega )$ and $H^*_{n}(\omega )$ is the inverse Fourier transform of $O_{n}(t)$ and the complex conjugate of the transfer function $H_{n}(\omega )$.

For the i^2^PIE technique, only a single scan is done to measure the spectrogram, after which the algorithm reconstructs the spectral phase and amplitude information.

### Spheroids of cardiomyoblast cells

For this work, rodent cardiomyoblast (H9c2) spheroids were grown, by seeding cells in a media (high glucose DMEM, 10% foetal bovine serum with 1% penicillin) with 2 nM Hoechst 33342. Spheroids were incubated at 37 ^∘^C at 5% CO_2_ and 70-80% humidity for 96 h. The spheroids were harvested by aspirating and setting the spheroid in 1% low-gelling agarose in fluorinated ethylene-propylene (FEP) tubing.

### Contrast measurements

Image contrasts were determined using Matlab. Images were converted to 8bit scales and scaled by a common factor with respect to the images acquired with i^2^PIE. For this work the contrast was calculated as^27^:

$$\begin{aligned} C_{\text {Weber}} = \frac{I_{\text {max}}-I_{\text {min}}}{I_{\text {background}}} \end{aligned}$$

Where $C_{\text {Weber}}$, $I_{\text {max}}$, $I_{\text {min}}$ and $I_{\text {background}}$ are the Weber contrast, maximum signal intensity, minimum signal intensity and background intensity respectively. The background intensity was taken as the mean background in the lower left of the image.

### Image processing

Image processing was done using ImageJ and Huygens deconvolution. Images were cropped and thresholds were set to remove backgrounds in ImageJ. All images were deconvolved using Huygens Professional version 23.10. The microscope parameters for the numerical aperture of the imaging system, the voxel sizes of the dataset ($340 \times 340 \times 500$ nm), the excitation and emission wavelengths of the system and the light-sheet thickness were set in Huygens software. A theoretical point spread function was then calculated by the Huygens deconvolution software and used to deconvolve the dataset using default parameters provided by the deconvolution wizard. The deconvolved data was further processed in ImageJ using a rolling ball background subtraction of 80 pixels and a LUT file was applied to add colour for display purposes.

## Data Availability

The datasets generated during and/or analysed during the current study are available from the corresponding author upon reasonable request.

## References

[1] Huisken J, Swoger J, Bene FD, Wittbrodt J, Stelzer EH (2004). Optical sectioning deep inside live embryos by selective plane illumination microscopy. Science.

[2] Icha J, Weber M, Waters JC, Norden C (2017). Phototoxicity in live fluorescence microscopy, and how to avoid it. BioEssays.

[3] Zipfel WR, Williams RM, Webb WW (2003). Nonlinear magic: Multiphoton microscopy in the biosciences. Nat. Biotechnol..

[4] Tang S, Krasieva TB, Chen Z, Tempea G, Tromberg BJ (2006). Effect of pulse duration on two-photon excited fluorescence and second harmonic generation in nonlinear optical microscopy. J. Biomed. Opt..

[5] Olarte OE (2012). Image formation by linear and nonlinear digital scanned light-sheet fluorescence microscopy with Gaussian and Bessel beam profiles. Biomed. Opt. Express.

[6] Hopt A, Neher E (2001). Highly nonlinear photodamage in two-photon fluorescence microscopy. Biophys. J..

[7] Maioli V (2020). Fast in vivo multiphoton light-sheet microscopy with optimal pulse frequency. Biomed. Opt. Express.

[8] Ji N, Magee J, Betzig E (2008). High-speed, low-photodamage nonlinear imaging using passive pulse splitters. Nat. Methods.

[9] Débarre D, Olivier N, Supatto W, Beaurepaire E (2014). Mitigating phototoxicity during multiphoton microscopy of live *Drosophila* embryos in the 1.0–1.2 $$\mu$$m wavelength range. PLoS One.

[10] Charan K, Li B, Wang M, Lin CP, Xu C (2018). Fiber-based tunable repetition rate source for deep tissue two-photon fluorescence microscopy. Biomed. Opt. Express.

[11] Heidt AM, Spangenberg D-M, Rampur A, Hartung A, Bartelt H (2022). All-Normal Dispersion Fiber Supercontinuum: Principles, Design, and Applications of a Unique White Light Source.

[12] Walowicz KA, Pastirk I, Lozovoy VV, Dantus M (2002). Multiphoton intrapulse interference. 1. Control of multiphoton processes in condensed phases. Phys. Chem. A.

[13] Lozovoy VV, Pastirk I, Walowicz KA, Dantus M (2003). Multiphoton intrapulse interference. II. Control of two- and three-photon laser induced fluorescence with shaped pulses. J. Chem. Phys..

[14] Lozovoy VV, Pastirk I, Dantus M (2004). Multiphoton intrapulse interference. IV. Ultrashort laser pulse spectral phase characterization and compensation. Opt. Lett..

[15] Comin A, Ciesielski R, Piredda G, Donkers K, Hartschuh A (2014). Compression of ultrashort laser pulses via gated multiphoton intrapulse interference phase scans. J. Opt. Soc. Am. B.

[16] Spangenberg D-M, Rohwer E, Brügmann M, Feurer T (2020). Extending time-domain ptychography to generalized phase-only transfer functions. Opt. Lett..

[17] Dwapanyin G (2020). Generalized spectral phase-only time-domain ptychographic phase reconstruction applied in nonlinear microscopy. J. Opt. Soc. Am. B.

[18] Viljoen R (2020). Implementation of temporal ptychography algorithm, i2PIE, for improved single-beam coherent anti-Stokes Raman scattering measurements. J. Opt. Soc. Am. B.

[19] Engelbrecht CJ, Stelzer EH (2006). Resolution enhancement in a light-sheet-based microscope (SPIM). Opt. Lett..

[20] Schwertner M, Booth M, Wilson T (2004). Characterizing specimen induced aberrations for high NA adaptive optical microscopy. Opt. Express.

[21] Xu C, Webb WW (1996). Measurement of two-photon excitation cross sections of molecular fluorophores with data from 690 to 1050 nm. J. Opt. Soc. Am. B.

[22] Olesiak-Banska J (2013). Revealing spectral features in two-photon absorption spectrum of Hoechst 33342: A combined experimental and quantum-chemical study. J. Phys. Chem. B.

[23] Dini S (2016). Identifying the necrotic zone boundary in tumour spheroids with pair-correlation functions. J. R. Soc. Interface.

[24] Dairkee SH, Deng G, Stampfer MR, Waldman FM, Smith HS (1995). Selective cell culture of primary breast carcinoma. Cancer Res..

[25] Lammerding J (2011). Mechanics of the Nucleus.

[26] Keller PJ, Stelzer EH (2010). Digital scanned laser light sheet fluorescence microscopy. Cold Spring Harbor Protoc..

[27] Pelli DG, Bex P (2013). Measuring contrast sensitivity. Vis. Res..

